# Impact of Varying Dietary Calcium Contents on the Gut Metabolomics of Yunnan Semi-Fine Wool Sheep (*Ovis aries*)

**DOI:** 10.3390/metabo14070381

**Published:** 2024-07-10

**Authors:** Muhammad Khan, Xiaoqi Zhao, Xiaojun Ni, Sikandar Ali, Baiji Danzeng, Hongyuan Yang, Maida Mushtaq, Jiachong Liang, Bai Xue, Guobo Quan

**Affiliations:** 1 Yunnan Animal Science and Veterinary Institute, Jindian, Panlong District, Kunming 650224, China; khanbwn011@gmail.com (M.K.); zhaoxiaoqi2023@163.com (X.Z.); nixiaojun123@126.com (X.N.); danzeng1376@163.com (B.D.); maomao7836@sohu.com (H.Y.); maidach.17@gmail.com (M.M.); ljj200311@163.com (J.L.); 2 Yunnan Provincial Engineering Research Center of Animal Genetic Resource Conservation and Germplasm Enhancement, Jindian, Panlong District, Kunming 650224, China; 3 Zhejiang Vegamax Biotechnology Co., Ltd., Huzhou 313300, China; sikandar.ptbav@gmail.com; 4 Animal Nutrition Institute, Sichuan Agricultural University, Wenjiang District, Chengdu 611137, China

**Keywords:** dietary calcium, metabolite, metabolic pathways, nutritional requirement, sheep

## Abstract

Yunnan semi-fine wool (YSFW) is a recently developed dual-purpose (meat and wool) sheep breed mainly found in Yunnan Province, China. Moreover, dietary calcium is essential for animal health and productivity. The current experiment aimed to investigate the impact of dietary calcium on sheep gut metabolite profile. For this, thirty YSFW rams (male, age = 10 months, and body weight = 40.37 ± 0.49 kg) were randomized into three groups (n = 10 rams/group), followed by a completely randomized design, and the groups were allotted to one of three dietary calcium levels (Q_1 = 0.50%, Q_3 = 0.73%, and Q_5 = 0.98% on a dry basis). The rams were fed ad libitum by feeding twice a day (at 08:00 and 17:00 h/day) throughout the experimental period (44 day). On the 21st day of the experiment, fecal samples were collected from 27 rams (9/group) and untargeted metabolite profiling was performed by using ultra-performance liquid chromatography. The PCA plot showed that the Q_5 group metabolites were clustered more tightly than for Q_1 and Q_3, respectively. The tightly clustering molecules were mainly alkaloids and their derivatives, benzenoids, lignans and related compounds, lipids, nucleotides, organic acids, and nitrogenous-based derivatives. According to the Kyoto Encyclopedia of Genes and Genomes pathway analysis, these molecules potentially contribute to metabolic pathways, biosynthesis of secondary metabolites, proteinaceous compounds, and the metabolism of the protein derivatives, particularly amino acids. The PLS-DA plots revealed a significant difference between the Q_1, Q_3, and Q_5 groups, suggesting that Q_5 had a clear separation across the groups. Based on the metabolomic analysis, feeding different levels of dietary calcium significantly changed the metabolomic profile of YSFW rams, which primarily entails metabolic pathways such as energy, protein, and lipid metabolism.

## 1. Introduction

Sheep are reared worldwide for milk, mutton, skin, and wool production. Currently, feeding systems particularly for fattening sheep are shifting toward precise feeding practices such as total mixed ration, as it provides balanced macro- and micronutrients in each bite to fulfill the sheep’s requirements [1]. An optimal calcium concentration in such a feeding system is essential, as it has diversified physiological roles such as bone and teeth formation, muscle contraction, blood coagulation, cell signaling, intestinal absorption mechanism, cellular membranous permeability, and hormone regulation [2,3]. The dietary calcium deficiency or imbalance leads to abnormal bone formation, hypocalcemia, and incoordination of muscles and nerves in animals [3,4,5]. Therefore, dietary calcium balancing is essential to smoothen the growth performance of the sheep. 

The factorial method [6,7,8] has been applied to fulfill the dietary calcium requirements of small ruminants. According to this method, the net requirements of animals are calculated by quantifying the calcium deposited in the body and conception products (fetus and associated materials) and excreted (milk and body wastes, like feces and urine). However, it is also well-established that several factors such as calcium source, dietary level, availability, and bioavailability from different sources also affect the net calcium requirements of sheep [7]. Furthermore, calcium regulation and its interactive impact with other minerals in the animal body is a complex mechanism [9] and is not fully understood. 

Mineral homeostasis regulation in ruminants is unique compared to monogastric species such as poultry and diversified even within ruminant species. For example, Böswald, Dobenecker [10] evaluated the dietary and fecal calcium levels of different ruminant species and they documented higher calcium absorption in hindgut fermenting species (*Diceros bicornis*, *Dicerorhinus sumatrensis*, *Rhinoceros unicornis*, *Equus caballus*, *Tapirus terrestris*, *Elephas maximus*, and *Oryctolagus cuniculus*) than domestic ruminants (*Bos taurus*, *Ovis aries*, and *Capra capra*). This higher calcium absorption in hindgut fermenter species removes excessive calcium from the intestine and might ensure higher availability for microbial fermentation [11]. The major excretory, as well as regulatory, route of calcium in hindgut fermenters, is the urine [11,12], while ruminants do not need to adopt such a bypass mechanism, as ruminal fermentation involves effectively recycling calcium into the rumen through saliva [13]. Calcium is well characterized regarding intestinal transportation and interactive ways with other minerals [5]. However, the transportation mechanism of calcium in the rumen has not been well defined until now. In addition, more integrated information regarding changes in the metabolomic profile of ruminants by modulating the dietary calcium levels will provide a better understanding of mineral homeostasis disturbance and such animals’ ability to adapt to marginal or excessive mineral supply. Therefore, the interplay between dietary calcium levels and metabolomic-based metabolism-regulating pathways was elucidated, as well as the key importance in meat- and milk-producing ruminants, particularly Yunnan semi-fine wool sheep. 

## 2. Materials and Methods

### 2.1. Experimental Station and Approval

This experiment was conducted on 23 December 2020 at the sheep farm of Kunming Yixingheng Livestock Technology Co., Ltd. (26°22′ N; 103°40′ E) after approval by the ethical committee of the Yunnan Animal Science and Veterinary Institute (202009002). The experimental procedures were carried out according to the approved protocols and guidelines of the State Science and Technology Commission of the People’s Republic of China, 1988, and the Standing Committee of Yunnan Provincial People’s Congress 2007.10). 

### 2.2. Research Design and Husbandry Practices

In this experiment, a total of thirty male Yunnan semi-fine wool rams (average age = 10 months and body weight = 40.37 ± 0.49 kg) were selected from the farm, randomly divided into three groups (n = 10 rams/group) following a completely randomized design, and groups were allotted to one of three dietary calcium levels (Q_1 = 0.50%, Q_3 = 0.73%, and Q_5 = 0.98%, respectively, on a dry basis). The total experimental duration was 44 days, including a 14-day dietary adaptation, followed by a 30-day feeding trial. The feed was offered twice/day (at 08:00 and 17:00 h/day) and 10% refusal was adjusted to ensure the ad libitum intake. The rams were given free access to fresh and clean water for 24 h/day. The isonitrogenous, isocaloric, and homogenous fiber content-containing diets are detailed in Table 1 regarding ingredient profile and chemical composition. The diet was formulated according to ruminants [7] to meet the nutrient requirements of 40 kg sheep with an average daily gain of 250 g/day. By considering ruminants’ [7] optimum dietary calcium recommendation (Q_3), upper (Q_5) and lower (Q_1) calcium levels were evaluated in this experiment. The chemical composition characteristics such as dry matter, crude protein, ether extract, and ash contents of the dietary treatments were determined by following the protocols of Cheung [14]. The contents of neutral detergent fiber and acid detergent fiber in dietary treatments were estimated using a fiber analyzer as per the established protocols of Van Soest [15].

### 2.3. Fecal Sample Collection and Metabolite Extraction

On the 21st day of the feeding trial, nine rams from each group were randomly subjected to fecal sampling for metabolomic analysis by following the sterile protocols detailed in our recent publication Ali, Ni [16]. Briefly, 10 g of feces was harvested directly from the terminal part of the rectum, transferred into a respective 10 mL sterile prelabeled tube (BIOFIL, Guangzhou, China), and immediately frozen at –80 °C in a liquid nitrogen-filled tank for further analysis. The preserved samples were transferred to the Hangzhou Lianchuan Biotechnology Co., Ltd., Hangzhou, China (HLBC) for metabolomic analysis by maintaining the cold supply chain. The sample preparation and metabolite extraction were carried out by conventional untargeted metabolomic protocols (LC-MS/MS) as detailed on the HLBC’s website. Briefly, 100 mg of fecal material from each sample was crushed in liquid nitrogen, then 20 µL of crushed fecal material was mixed with 120 µL pre-cooled methanol (50% at −20 °C), vortexed for 1 min, incubated for 10 min at room temperature, refrigerated (−20 °C) overnight for precipitation, and then supernatants were transferred into 96-well plates after centrifugation at 4000× *g* for 20 min. A 10 µL portion of crushed fecal material from each sample was also mixed in parallel to make the pooled quality control (QC) sample. The prepared plates were kept at −80 °C before loading in the LC-MS machine by following the acquired order of the system. The ultra-performance liquid chromatography (UPLC) system 162 (SCIEX, Macclesfield, UK) was used for chromatographic separations by using ACQUITY UPLC T3 column (100 mm * 2.1 mm, 1.8 µm, Waters, Wilmslow, UK) at 0. 164 4 mL/min flow rate for reversed-phase separation. The column oven temperature was maintained at 35 °C. Solvent A (water, 0. 1% formic acid) and solvent B (phenol, 0.1% formic acid) were used in an equal ratio to make the mobile phase. The system was adjusted for gradient elution conditions at 0–0.5 min, 5% B; 0.5~7 min, 5% to 100% B; 7~8 min, 100% B; 8–8.1 min, 100% to 5% B; 8.1–10 min, 5% B with an injection volume of 4 µL. The high-resolution tandem mass spectrometer TripleTOF5600plus (SCIEX, Macclesfield, UK) was used to detect the column’ eluted metabolites. The QTOF operation was adjusted to operate in positive and negative ion modes. The gas pressures were adjusted to 30 PSI and 60 PSI for curtain gas and ion source gases, respectively. The temperature of the interface heater was adjusted at 650 °C with 5000 V 172 and 4500 V ion spray voltage for positive and negative ion modes, respectively. The 60 to 1200 Da IDA and TOF’s mass range was adjusted in data collection mode. In the plus one charge state, approximately 12 product ion scans over counts per second were obtained during the 150 ms survey scans. A 40 GHz multichannel TDC detector with four anode/channel detectors was monitored by four-time bins summing each scan by changing the pulse frequency at 11 kHz. The overall cycle time was set at 0.56 s. Four seconds was the dynamic exclusion timer’s setting. Throughout the acquisition, the mass accuracy was calibrated after every 20 samples. In addition, after 180, every 10 samples, a quality control sample (pool of all samples) was obtained to assess the stability of the LC-MS during the acquisition.

### 2.4. Bioinformatics and Statistical Analysis

The MS data file was processed with XCMS software (3.19 version), which includes processes for peak selection, peak grouping, retention time correction, second peak grouping, and isotope and adduct annotation. The raw LC-MS data were transformed to mzXML format using R studio tools including XCMS, CAMERA, and MetaX. Ionic identification uses both retention time (RT) and *m*/*z* values. A three-dimensional matrix was generated by assigning peak indices (retention duration and *m*/*z* value pairs), sample names (observations), and ion intensity data (variables). Metabolites were annotated by comparing the exact molecular mass (*m*/*z*) of samples to those in the Kyoto Encyclopedia of Genes and Genomes (KEGG) pathways and Human Metabolome Database (HMDB) databases, with a mass difference of less than 10 ppm. The molecular formulae were determined and validated using isotopic distribution values. Additionally, an in-house fragment spectrum library was employed to validate metabolite identification. MetaX was used for further processing peak data intensity. To improve the data quality, features found in less than half of QC samples or 80% of biological samples were deleted, and the remaining peaks with missing values were processed using the k-nearest neighbor approach. The preprocessed dataset was then analyzed using PCA to identify outliers and assess batch effects. To reduce signal intensity drift over time, a strong LOESS signal correction was applied to QC data based on injection order. Metabolic characteristics with relative standard deviations larger than 30% in all QC samples were excluded. The statistical significance difference among results was tested by using a permutational multivariate ANOVA test. The generalized linear models of R studio were used for normalized individual metabolite abundances (counts per million) among lamb groups. Significant differences were found with a *p*-value of <0.01.

## 3. Results

### 3.1. Fecal Metabolite Identification, Quantification, and Annotation

**Figure 1:**
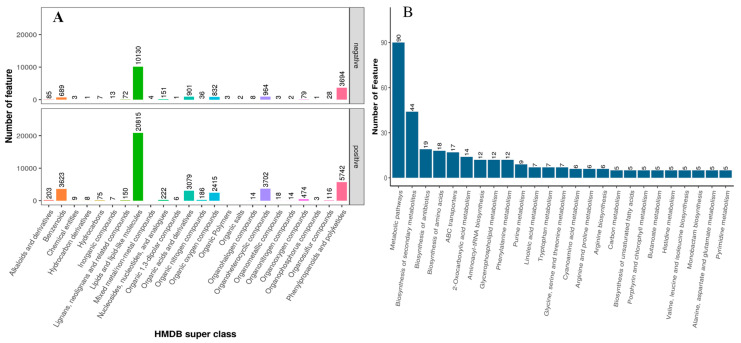
The HMBD superclass (**A**) and KEGG pathway (**B**) analysis of detected metabolites and top 30 associated metabolic pathways in positive and negative ionic modes, respectively.

The collected fecal samples of Yunnan semi-fine wool rams fed different dietary calcium levels were subjected to the UHPLC for the untargeted metabolite screening. In this study, the total detection was 12,528, including 7084 and 5474 in positive and negative ionic modes, respectively (Figure 1A). For MS2, HMBD, and KEGG levels, the detected molecules were 783 (containing 445 positives and 338 negative ionic modes), 6849 (including 4420 positive and 2429 negative ionic modes), and 6006 (including 3848 positive and 2158 negative ionic modes, respectively). Moreover, the annotated molecules’ count was 8090 (including 4945 positive and 3146 negative ionic modes).

### 3.2. Metabolomic Profiling

**Figure 2:**
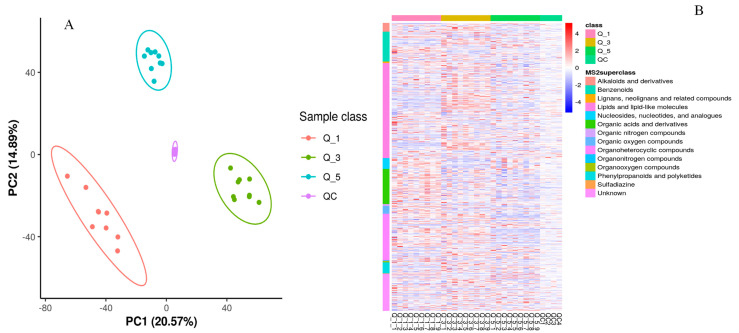
The quantification analysis of identified metabolites by PCA analysis (**A**) and heatmaps (**B**) for different groups (red = Q_1, green = Q_3, and blue = Q_5).

In the PCA and KEGG pathways analysis (Figure 2), there is a clear variation regarding metabolite enrichment in the fecal samples of rams fed different levels of dietary calcium. According to Figure 2A, the Q_5 group had a tighter aggregation of dots and clearer segregation than Q_3 and Q_1, respectively. The tight aggregation of the dots in the PCA plot for a treatment group represents similarity. In contrast, groups cleared parting from each other, meaning that there was a significant difference regarding metabolite profile among groups. These results suggest that dietary calcium levels significantly impacted the fecal metabolite profile of the rams and the Q_5 group had clear demarking metabolite profiles across the groups. Similarly, heatmap analysis (Figure 2B) revealed that the Q_5 group had more enrichment of the metabolites than the Q_3 and Q_1 groups, respectively. The tightly clustering molecules were mainly alkaloids and their derivatives, benzenoids, lignans and related compounds, lipids, nucleotides, organic acids, and nitrogenous-based derivatives. According to the KEGG pathway analysis (http://www.genome.jp/kegg/ accessed on 1 March 2021), these molecules potentially contribute to metabolic pathways and biosynthesis of secondary metabolites, proteinaceous compounds, and the metabolism of the protein derivatives, particularly amino acids (Figure 1B). So far, these results indicate that dietary calcium levels had a significant role in the growth and production performance of the Yunnan semi-fine wool rams.

### 3.3. Differential Metabolomic Profiling

**Figure 3:**
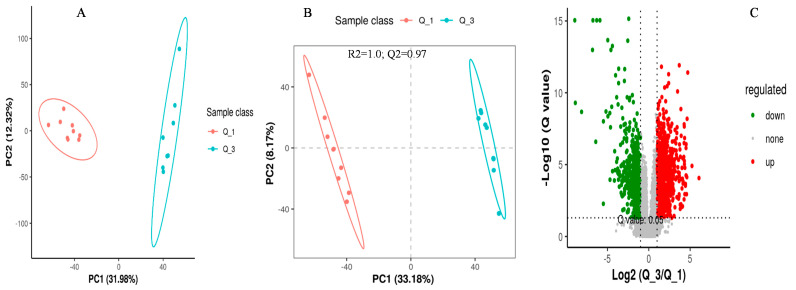
The comparative discriminated metabolomics plotting ((**A**) = PCA plot, (**B**) = PLSDA plot, and (**C**) = volcano plot, respectively) for the Q_1/Q_3 groups.

**Figure 4:**
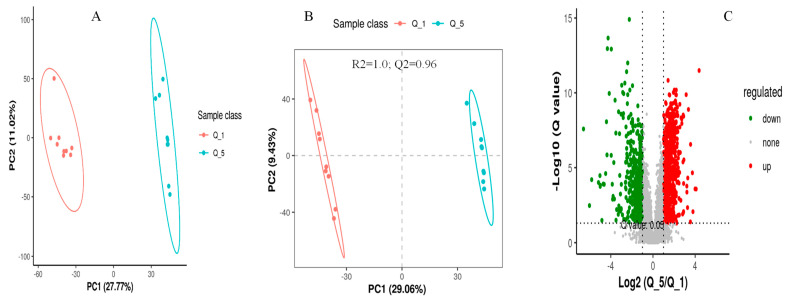
The comparative discriminated metabolomics plotting ((**A**) = PCA plot, (**B**) = PLSDA plot, and (**C**) = volcano plot, respectively) for the Q_1/Q_5 groups.

**Figure 5:**
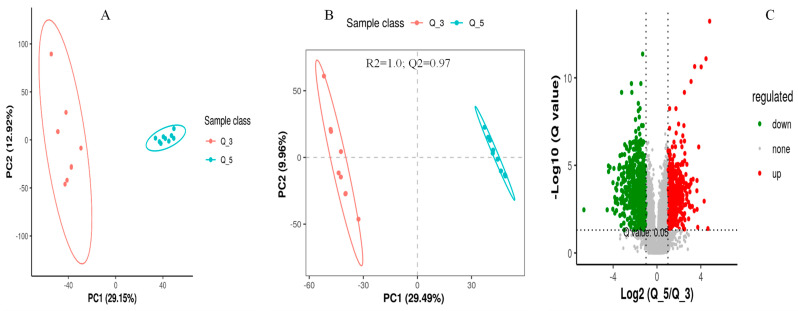
The comparative discriminated metabolomics plotting ((**A**) = PCA plot, (**B**) = PLSDA plot, and (**C**) = volcano plot, respectively) for the Q_3/Q_5 groups.

The screening of differential metabolites among different dietary groups was carried out by setting the statistical standards at a q-value of 0.5 and ratio > 2, as presented in the PCA, PLS-DA, and volcano plots, respectively (Figure 3, Figure 4 and Figure 5). According to the PSL-DA analysis, the pairwise comparison for discriminated metabolites among different treatment groups had significant metabolomic differences (Figure 3B, Figure 4B, and Figure 5B). The PLS-DA is a powerful tool for removing the noncorrelated variations in spectra before the discrimination of the ionic peaks, and thus, it helps to detect the potential biomarkers in comparative analysis. The PLS-DA plots revealed a significant difference between the Q_1, Q_3, and Q_5 groups and intercepts (R2 and Q2), suggesting that Q_5 had more precise as well as clear separation across the groups (Figure 3B, Figure 4B, and Figure 5B). The Q2 values were observed to be less than zero with a minimum of 1, indicating that the model was not overfitted (Figure 3B, Figure 4B, and Figure 5B). Similarly, the observed Q2 values (ranging between 0.96 and 0.97) suggest that the model was not overfitted (Figure 3B, Figure 4B, and Figure 5B). The volcano plots (Figure 3C, Figure 4C, and Figure 5C) suggest that Q_5 had clear segregation and regulation patterns across the treatments.

### 3.4. Pairwise Comparison of Discriminated Metabolites

The statistical significance (*p*-value), coefficient of variation (CV), VIP scoring, and regulation type (either up or down) of discriminated metabolites (pairwise comparisons from both positive as well as negative ionic modes) are listed in Table 2, Table 3, Table 4, Table 5, Table 6, Table 7 and Table 8. In differential analysis, a total of 149 metabolites were significantly altered across all possible pairs. The detected molecules were 20, 28, and 16 for Q_1/Q_3, Q_1/Q_5, and Q_3/Q_5 groups, respectively. The shared molecules between different groups were 34 (between Q_1/Q_3 and Q_1/Q_5 groups), 21 (between Q_1/Q_3 and Q_3/Q_5 groups), 21 (between Q_1/Q_5 and Q_3/Q_5 groups), and 13 (between Q_1/Q_3 and Q_1/Q_5, and Q_3/Q_5 groups). In the pairwise comparison of Q_1/Q_3, the alkaloids and their derivatives, nucleosides/nucleotides and their analogs, organic acids and their derivatives, and organic oxygen compounds showed downregulation (Table 2). In contrast, phenylpropanoids and polyketides showed upregulation. However, lipid and lipid molecules and organoheterocyclic compound-based metabolites showed up- and downregulation patterns. The pair of Q_1/Q_5 showed upregulation for the superclass of the lipid and lipid molecules, benzenoids, organic acids and their derivatives, phenylpropanoids and polyketides, and unknown superclass (Table 3). However, the superclass of the alkaloids and their derivatives, organic oxygen compounds, and organoheterocyclic compounds showed downregulation. According to Table 4, the alkaloids and their derivatives, lipid and lipid molecules, organic acids and their derivatives, and organoheterocyclic compound superclass were downregulated for the pair of Q_3/Q_5. However, lipid and lipid molecules, benzenoids, and organic acids and their derivatives showed up- and downregulation patterns.

### 3.5. Metabolomic Analysis of Mutually Shared Discriminated Metabolites between Different Pairs

Table 5, Table 6, Table 7 and Table 8 list the mutually shared discriminated metabolites between all possible pairs. These mutually shared metabolites between pairs (Q_1/Q_3, Q_1/Q_5, and Q_3/Q_5) showed significant differences in the fecal metabolite profiling. The metabolite regulation pattern regarding the comparison of all three pairs (Q_1/Q_3, Q_1/Q_5, and Q_3/Q_5) showed clear differences for mutually shared discriminated metabolites (Table 8) and suggested that Q_3/Q_5 had reciprocal regulation compared with other groups (Table 5). According to Table 6, the regulation pattern of mutually shared metabolites for the listed superclass in the groups (Q_1/Q_3 and Q_1/Q_5) was changed significantly, without any individual metabolite variation. However, according to Table 6, the regulation pattern of mutually shared individual metabolites between the Q_1/Q_3 and Q_3/Q_5 pairs was reciprocal to each other. We observed that those metabolites that were downregulated for the Q_3/Q_5 pair were upregulated for the Q_1/Q_3 pair and vice versa (Table 6). The comparison of the Q_1/Q_5 and Q_3/Q_5 pairs revealed that only two mutually shared molecules (2-hydroxy stearate and 3-phenyl propionyl-glycine) had variations in their regulation pattern and both were upregulated in the former pair (Table 7). The differential detection of discriminated metabolites in this experiment was mainly associated with metabolism pathways, biosynthesis of secondary metabolism, and biosynthesis of antibiotics and amino acids. These results suggest that the Q_5 group had greater changes across the groups regarding fecal metabolic profile, particularly associated with metabolic pathways and biosynthesis processes.

## 4. Discussion

Micronutrients, like minerals and vitamins, perform several vital physiological and metabolic actions in animals’ bodies, resulting in better health and performance. Calcium is an indispensable major mineral with diverse metabolic roles, ranging from supporting structural functions in bones/teeth to empowering several physiological processes like muscle contraction, cellular signaling, neurotransmitter release, enzyme activation, hormonal regulation, and blood clotting [17]. Several studies documented that dietary calcium levels directly influence feed intake, body structure development, growth performance, and feed efficiency of the sheep [4,18]. Thus, adequate dietary calcium supply is essential for optimal metabolic functioning and preventing calcium deficiency-associated metabolic disorders. According to our knowledge, data regarding how dietary calcium levels can influence metabolic pathways are still scarce. Therefore, in this experiment, the fecal metabolomic profile of the Yunnan semi-fine wool rams fed different dietary calcium levels was investigated using LC-MS analysis. LC-MS is a widely used well-known technique across the globe for metabolomic profiling due to its accuracy, higher sensitivity, and precise analysis within a short time [19]. According to the PLS-DA analysis, the fecal metabolomic profile was significantly changed across the groups, suggesting an impact of dietary calcium levels on rumen fermentation and gut microbial composition. Previous research revealed that dietary modulation regarding nutrients has a direct influence on the gut microbiota composition of ruminants [20,21]. For example, changing dietary selenium and chromium contents results in a change in sheep’s gut microbiota diversity [21,22,23]. The gut microbiota has a versatile nature, adjusting in accordance with the diet, physical environment, and health status of the animal [11,23], and thus, changing the fecal metabolomic profile in this experiment reflects the dietary calcium influence on gut microbiota composition [24]. As dietary calcium influences the gut microbiota, this suggests that calcium can alter the fermentation process, leading to changes in the production of short-chain fatty acids and other metabolites. Studies documented that changing dietary calcium levels results in variation in the molar ratio of the rumen short-chain fatty acids [25,26,27]. So far, higher calcium levels might smoothen the microbial fermentation efficacy by changing their composition and thus ultimately alter the fecal metabolomic profile of rams. In the current study, a total of 149 differential metabolites were identified that were significantly regulated (up/down) across the treatment groups. Interestingly, most of these metabolites were upregulated for the Q_1 and Q_5 groups and downregulated for the Q_3 group. The differential detection of discriminated metabolites in this experiment was mainly associated with metabolic pathways (mainly energy, protein, and fatty acids), biosynthesis of secondary metabolism, and biosynthesis of antibiotics and amino acids. The trend lines of differential metabolites among groups demonstrated a higher regulation of metabolic pathways, with notable effects on purine, alanine, tryptophan, and glucose metabolism. These results underscore the multifaceted role of dietary calcium in modulating metabolic processes. Dietary calcium’s impact on purine metabolism, which is essential for numerous biochemical processes and their metabolism, is crucial for maintaining cellular energy homeostasis [28]. The regulation of alanine and tryptophan metabolism further underscores the importance of calcium in protein synthesis and neurotransmitter production [29]. Alanine plays a critical role in glucose metabolism and energy production, while tryptophan is a precursor for serotonin and melatonin [30]. These findings suggest that dietary calcium might enhance the overall protein turnover and neurotransmitter synthesis, contributing to better health and physiological functions in rams. The lower gut microbiota metabolized the primary bile acids into secondary metabolites [31]. It has been established by previous research that bile acids are involved in fat metabolism [32] and cellular proliferation, particularly intestinal, by modulating gene expression [33]. Moreover, it regulates the bioenergetics of glucose and lipids in the intestine, the liver, muscles, and adipose tissues [34]. Enhanced regulation in this pathway implies that dietary calcium could play a role in modulating the stress response and maintaining metabolic homeostasis. In this way, bile acids have a meaningful role in bioenergetics, lipoprotein metabolism, lower gut integrity, and immunity [35]. The higher dietary calcium levels might bind bile acids [36] and thus in this way can influence the secondary metabolite profile in the feces. 1,1’-[1,12-Dodecanediylbis(oxy)] bis benzene is an intermediate molecule that is produced during microbial degradation of dietary ingredients. Benzidine is another molecule that is downregulated with increasing dietary calcium levels. Calcium involves many biological activities, including enzyme function [37]. While it does not directly modulate cytochrome P450 enzymes (which metabolize citalopram), changing calcium homeostasis may have an indirect impact on liver function and enzyme activity [38]. Similarly, dietary calcium level has a direct impact on the absorption and excretion of other minerals, particularly associated with electrolyte balancing [39]. It is well known that changes in electrolyte balance result in compromised gut health and altered fecal metabolomic profile [24,40]. Calcium is essential for maintaining mineral and electrolyte balance., The observed changes in the metabolite profile indicate that dietary calcium plays a pivotal role in ensuring the proper balance of minerals and electrolytes, which is vital for the overall metabolic health of rams. The changes in protein metabolism in the current experiment can be associated with calcium interactive effects. It is well established that calcium binds with dietary protein to form the insoluble complex to aid their breakdown and fermentation, which results in a change in the dietary protein utilization [41]. Alteration in dietary protein utilization and fermentation patterns can transform the profile of the fecal nitrogenous metabolites. Enhanced regulation of nitrogen metabolism pathways indicates improved protein turnover and waste excretion [42]. Nitrogen balance is crucial for growth and maintenance, and its efficient regulation can lead to better protein utilization and reduced metabolic waste [16,43]. By influencing the pathways involved in lipid synthesis and breakdown, dietary calcium can help maintain lipid balance, which is essential for energy storage and membrane integrity. Calcium’s soapy form conjugated with fatty acids in this way impacts the overall lipid metabolism [44] and increases the fatty acid excretion in feces.

## 5. Conclusions

The fecal metabolomic analysis indicated that dietary calcium led to a high abundance of metabolites, which primarily entails metabolic pathways involved in energy production, lipid metabolism, and protein metabolism. This includes increased metabolites associated with fatty acid metabolism, and pathways critical for protein biosynthesis. Enhanced levels of fatty acid metabolites indicate increased lipid metabolism, supporting cellular energy needs. These findings underscore the role of dietary calcium in modulating various metabolic processes, reflecting its importance in overall metabolic health and function. These findings also support that dietary calcium can smoothen the growth and health of ruminants, particularly sheep, by one or a combination of the following metabolic pathways: (1) enhancing the protein availability for co-factors and immunity organ synthesis, (2) producing volatile fatty acids for bioenergetic processes, (3) maintaining mineral homeostasis, and (4) modulating the gut metabolic process to enhance the production of metabolic enzymes. However, the effect of dietary calcium content on gut microbiota profile in sheep nutrition has not been completely investigated, which is a limitation of the current study. As a result, additional research is needed to understand how dietary calcium content affects ruminant performance by changing gut microbiota composition utilizing advanced sequencing technologies.

## Figures and Tables

**Table 1:** Dietary formulations and chemical composition on a dry basis.

List of Ingredients	Inclusions		
	Q_1	Q_3	Q_5
Corn silage (%)	12.60	27.00	30.00
Wheat straw (%)	20.00	22.50	18.00
Corn grains (%)	28.00	15.00	13.50
Cornstarch (%)	12.60	16.60	14.90
Broad bean stalk powder (%)	16.00	0.00	2.00
Soybean meal (%)	9.15	10.00	10.20
Rapeseed meal (%)	0.00	4.30	1.60
Wheat bran (%)	0.00	2.50	6.95
Mineral and vitamin premix ^1^ (%)	1.00	1.00	1.00
Calcium carbonate (%)	0.00	0.50	1.35
Calcium hydrogen phosphate (%)	0.00	0.10	0.00
Sodium dihydrogen phosphate (%)	0.15	0.00	0.00
Salt (%)	0.20	0.50	0.50
Baking soda (%)	0.30	0.00	0.00
**Nutrient contents**			
ME ^2^ (MJ/kg)	9.31	9.30	9.30
CP ^3^ (%)	10.49	10.45	10.42
NDF ^4^ (%)	34.12	34.47	34.31
ADF ^5^ (%)	19.60	19.43	18.61
Ca (%)	0.50	0.73	0.98
P (%)	0.28	0.31	0.31

^1^ The premix provided the following per kg of diets: Mn 58 mg, Fe 145 mg, Zn 80 mg, Cu 10 mg, I 2.5 mg, Se 0.35 mg, Co 0.65 mg, VA 10,000 IU, VD3 1000 IU, VE 50 IU. ^2^ ME was calculated by NRC-2007 equations, while the others were measured values. ME: metabolic energy, ^3^ CP: crude protein, ^4^ NDF: neutral detergent fiber, and ^5^ ADF: acid detergent fiber.

**Table 2:** Comparative fecal metabolite regulation (up/down) of the Q_1/Q_3 groups of the Yunnan semi-fine wool rams fed different levels of dietary calcium.

Superclass	Metabolite	*p*-Value	VIP	CV	Regulated
Alkaloids and derivatives	Gelsemine	0.03	1.36	0.22	down
Lipids and lipid-like molecules	3-Methyladipic acid	0.00	1.22	0.01	down
	Anhydrocinnzeylanol	0.00	1.65	0.04	down
Nucleosides, nucleotides, and analogues	Cyclic AMP	0.00	1.55	0.05	down
Organic acids and derivatives	L-Phenylalanine	0.00	2.74	0.24	down
Organic oxygen compounds	7-Methylthioheptyl glucosinolate	0.00	1.74	0.03	down
	Methylbutanal	0.01	1.35	0.02	down
Organoheterocyclic compounds	Chrysanthemolactone	0.00	1.57	0.43	down
	Iressa	0.00	1.46	0.22	down
Unknown	3-Hydroxybenzaldehyde	0.00	1.86	0.03	down
Lipids and lipid-like molecules	Acylcarnitine 19:3	0.00	2.15	0.11	up
	Androstenedione	0.01	1.36	0.28	up
	Exemestane	0.00	1.71	0.10	up
	Isopropyl tiglate	0.03	1.40	0.04	up
	Oleamide	0.01	1.31	0.16	up
	Ursocholic acid	0.00	2.17	0.02	up
Organoheterocyclic compounds	alpha-Methyl-2-furanacrolein	0.01	1.23	0.33	up
	Chlordiazepoxide	0.00	1.68	0.09	up
Phenylpropanoids and polyketides	8-Carboxy-3-methylflavone	0.00	1.67	0.21	up
Unknown	Irgarol	0.01	1.54	0.19	up

**Table 3:** Comparative fecal metabolite regulation (up/down) of the Q_1/Q_5 groups of the Yunnan semi-fine wool rams fed different levels of dietary calcium.

Superclass	Metabolite	*p*-Value	VIP	CV	Regulated
Alkaloids and derivatives	6-allyl-8b-Carboxy-ergoline	0.00	1.84	0.24	down
Organic oxygen compounds	Erythrose	0.00	2.15	0.04	down
Organoheterocyclic compounds	1H-Indole-3-propanoic acid	0.00	1.76	0.07	down
	Piperidine	0.00	1.78	0.26	down
	Thymine	0.00	2.08	0.03	down
Unknown	2,2-Dimethylglutaric acid	0.00	2.14	0.05	down
	Allose	0.00	2.16	0.05	down
	Cyproconazole	0.01	1.80	0.12	down
Alkaloids and derivatives	Baptifoline	0.00	2.17	0.05	up
Benzenoids	Ginkgolic acid I	0.00	1.73	0.25	up
Lipids and lipid-like molecules	11′-Carboxy-gamma-chromanol	0.00	1.99	0.11	up
	12-Hydroxy-8,10-octadecadienoic acid	0.00	1.74	0.09	up
	16-Hydroxyhexadecanoic acid	0.00	1.36	0.04	up
	PGH3	0.00	2.02	0.08	up
	Prostaglandin A2	0.02	1.95	0.13	up
	Prostaglandin D3	0.02	1.82	0.17	up
	Sesquisabinene hydrate	0.00	2.13	0.13	up
Organic acids and derivatives	5-Hydroxyindoleacetylglycine	0.00	2.00	0.13	up
Phenylpropanoids and polyketides	8-Carboxy-3-methylflavone	0.00	2.30	0.21	up
	Hydrocinnamic acid	0.12	1.29	0.04	up
Unknown	17.alpha.-Dihydroequilin	0.00	1.74	0.14	up
	Artemisinin	0.00	1.81	0.07	up
	Cardamomin	0.00	2.20	0.15	up
	Diethyl sebacate	0.07	1.76	0.04	up
	Pheophorbide a	0.00	2.15	0.05	up

**Table 4:** Comparative fecal metabolite regulation (up/down) of the Q_3/Q_5 groups of the Yunnan semi-fine wool rams fed different levels of dietary calcium.

Superclass	Metabolite	*p*-Value	VIP	CV	Regulated
Alkaloids and derivatives	10-alpha-methoxy-9,10-dihydrolysergol	0.01	2.75	0.07	down
	Hirsuteine	0.00	1.63	0.14	down
Benzenoids	10-Gingerol	0.01	1.78	0.08	down
Lipids and lipid-like molecules	Ecabet	0.02	1.68	0.17	down
Organic acids and derivatives	2-Oxopentanedioic acid	0.00	1.88	0.08	down
Organoheterocyclic compounds	Eletriptan	0.02	1.55	0.15	down
	Norfloxacin	0.00	2.22	0.10	down
	Sclareolide	0.08	1.49	0.13	down
Benzenoids	4-Methylhippuric acid	0.00	1.97	0.03	up
Lipids and lipid-like molecules	Acylcarnitine 11:0	0.02	1.59	0.20	up
	Dodecanedioic acid	0.00	1.96	0.13	up
	Tetradecanedioic acid	0.01	1.56	0.15	up
	Traumatin	0.00	2.12	0.04	up
Organic acids and derivatives	N-Acetylproline	0.00	1.77	0.08	up
	Tiglylglycine	0.00	2.02	0.08	up
Unknown	Dimethyl sebacate	0.00	1.87	0.07	up

**Table 5:** Comparative regulation (up/down) of the mutually shared metabolites between Q_1/Q_3 and Q_1/Q_5 groups of the Yunnan semi-fine wool rams fed different levels of dietary calcium.

Superclass	Metabolite	Q_1/Q_3				Q_1/Q_5			
		*p*-Value	VIP	CV	Regulated	*p*-Value	VIP	CV	Regulated
Alkaloids and derivatives	Harman	0.00	1.53	0.07	up	0.00	2.75	0.07	up
Benzenoids	2-acetoxy-4-pentadecylbenzoic acid	0.00	2.38	0.04	up	0.02	1.49	0.04	up
	2-acetoxy-6-pentadecylbenzoic acid	0.00	2.04	0.15	up	0.00	1.45	0.15	up
	2-Hydroxy-3-methoxy benzaldehyde	0.02	2.24	0.06	down	0.02	2.67	0.06	down
	4-Methylhippuric acid	0.00	3.75	0.03	down	0.00	2.26	0.03	down
	Ortho-Hydroxyphenylacetic acid	0.00	2.25	0.09	down	0.00	1.71	0.09	down
	p-Cresol	0.00	2.23	0.05	down	0.00	1.75	0.05	down
	Phenylacetic acid	0.01	1.74	0.05	up	0.03	1.43	0.05	up
	p-Tolyl phenylacetate	0.00	1.55	0.02	up	0.00	2.97	0.02	up
Lipids and lipid-like molecules	18-Hydroxyeicosatetraenoic acid	0.00	2.39	0.10	up	0.00	2.14	0.10	up
	Acetyl-DL-carnitine	0.00	1.69	0.15	down	0.00	1.76	0.15	down
	Acylcarnitine 19:4	0.00	2.16	0.13	up	0.00	1.73	0.13	up
	Adipic acid	0.39	1.77	0.02	up	0.09	1.78	0.02	up
	Cinobufagin	0.00	1.67	0.12	up	0.02	2.19	0.12	up
	Ethyl icosapentate	0.00	1.68	0.02	up	0.00	2.09	0.02	up
Nucleosides, nucleotides/analogue	Deoxyinosine	0.00	1.49	0.50	down	0.00	1.94	0.50	down
Organic acids and derivatives	Creatine	0.00	2.57	0.03	down	0.00	2.63	0.03	down
	o-Phenanthroline	0.00	1.60	0.07	up	0.00	2.88	0.07	up
	Tyr-Leu	0.00	1.96	0.06	up	0.00	2.15	0.06	up
Organic oxygen compounds	Trehalose	0.00	1.37	0.04	down	0.00	2.40	0.04	down
Organoheterocyclic compounds	Amlexanox	0.00	1.54	0.05	up	0.00	3.01	0.05	up
	Anileridine	0.08	1.49	0.10	up	0.01	1.39	0.10	up
	Deoxydihydro-artemisinin	0.00	1.45	0.03	up	0.00	2.27	0.03	up
	Pyrophaeophorbide a	0.00	1.74	0.05	up	0.00	2.12	0.05	up
	Sempervirine	0.00	1.51	0.07	up	0.00	2.19	0.07	up
Phenylpropanoids and polyketides	alpha,beta-Dihydroresveratrol	0.00	1.33	0.22	up	0.00	2.78	0.22	up
	beta-Zearalenol	0.00	1.48	0.03	up	0.00	2.10	0.03	up
	Gerberinol	0.00	2.29	0.04	down	0.00	2.16	0.04	down
	Moracin G	0.00	1.35	0.03	up	0.00	2.76	0.03	up
Unknown	2-Indolinone	0.01	1.97	0.06	up	0.00	2.31	0.06	up
	3’-Deoxyguanosine	0.00	1.93	0.15	down	0.00	1.61	0.15	down
	4-Coumaroylcholine	0.00	2.20	0.13	down	0.00	2.92	0.13	down
	S-Hydroprene	0.01	1.69	0.33	up	0.09	1.41	0.33	up
	Xanthene-9-carboxylic acid	0.00	1.47	0.01	up	0.00	2.79	0.01	up

**Table 6:** Comparative regulation (up/down) of the mutually shared metabolites between Q_1/Q_3 and Q_3/Q_5 groups of the Yunnan semi-fine wool rams fed different levels of dietary calcium.

Superclass	Metabolite	Q_1/Q_3				Q_3/Q_5			
		*p*-Value	VIP	CV	Regulated	*p*-Value	VIP	CV	Regulated
Alkaloids and derivatives	Pilocarpine	0.00	2.60	0.07	up	0.00	2.41	0.07	down
Benzenoids	3-Ethylphenol	0.01	1.58	0.07	up	0.02	1.40	0.07	down
	3-Hydroxyanthranilic acid	0.00	1.53	0.09	up	0.00	2.79	0.09	down
	3-Methylbenzaldehyde	0.01	1.63	0.09	up	0.02	1.48	0.12	down
	Isohomovanillic acid	0.00	1.51	0.27	up	0.00	2.17	0.27	down
Lipids and lipid-like molecules	12-Ketodeoxycholic acid	0.00	2.49	0.11	up	0.00	1.55	0.07	down
	20-Hydroxyarachidonic acid	0.09	1.29	0.16	up	0.07	1.39	0.16	down
	2-Isopropylmalic acid	0.00	1.86	0.24	up	0.00	2.00	0.24	down
	7C-aglycone	0.01	2.01	0.43	up	0.07	1.76	0.43	down
	Acylcarnitine 22:1	0.00	1.73	0.25	up	0.00	1.57	0.25	down
	Deoxycholic acid	0.00	2.18	0.10	up	0.00	2.20	0.06	down
	Ethylmalonic acid	0.00	1.30	0.20	up	0.01	2.05	0.20	down
	Hyocholic acid	0.00	2.46	0.28	up	0.00	1.88	0.28	down
	Pelargonic acid	0.00	1.68	0.06	up	0.00	2.13	0.06	down
	Ricinoleic acid	0.03	1.52	0.03	up	0.07	1.30	0.03	down
Nucleosides, nucleotides, and analogs	Guanosine	0.00	1.87	0.21	down	0.00	1.75	0.21	up
	Inosine	0.00	1.89	0.21	down	0.03	1.29	0.21	up
Organoheterocyclic compounds	2-Indolecarboxylic acid	0.00	1.57	0.03	down	0.00	2.93	0.03	up
	4-Hydroxyquinoline	0.00	1.51	0.15	up	0.07	1.86	0.05	up
	Quinoline-2,6-diol	0.00	1.45	0.06	down	0.00	2.83	0.06	up
Phenylpropanoids	p-Coumaraldehyde	0.01	1.56	0.05	up	0.02	1.60	0.05	down

**Table 7:** Comparative regulation (up/down) of the mutually shared metabolites between Q_1/Q_5 and Q_3/Q_5 groups of the Yunnan semi-fine wool rams fed different levels of dietary calcium.

Superclass	Metabolite	Q_1/Q_5				Q_3/Q_5			
		*p*-Value	VIP	CV	Regulated	*p*-Value	VIP	CV	Regulated
Benzenoids	2-Phenylethanaminium	0.00	2.70	0.08	up	0.00	2.58	0.08	up
	4-Methylcatechol	0.00	2.66	0.02	down	0.07	1.44	0.02	down
	Styrene	0.00	2.86	0.05	up	0.00	2.94	0.05	up
Lipids and lipid-like molecules	(9S,10S)-9,10-dihydroxyoctadecanoate	0.00	1.71	0.03	down	0.00	1.52	0.03	down
	13’-Carboxy-alpha-tocotrienol	0.00	1.50	0.03	down	0.00	2.52	0.03	down
	2-hydroxy stearate	0.00	1.91	0.27	down	0.00	2.04	0.04	up
	Glycocholic acid	0.00	1.90	0.06	up	0.00	2.65	0.06	up
Organic acids and derivatives	(3-Phenylpropionate)glycine	0.00	1.92	0.15	down	0.00	1.81	0.15	up
	3-Hydroxyglutaric acid	0.00	2.75	0.04	down	0.00	1.63	0.04	down
Organic nitrogen compounds	Spermidine	0.00	1.97	0.21	up	0.00	2.34	0.21	up
Organoheterocyclic compounds	Indole-3-propionic acid	0.00	1.82	0.07	down	0.00	1.77	0.07	down
	Isokessane	0.01	1.56	0.06	up	0.00	2.51	0.06	up
	Koumine	0.09	1.79	0.06	up	0.02	1.54	0.09	up
	Kynurenic acid	0.00	1.76	0.04	down	0.00	2.02	0.03	down
	Phaeophorbide b	0.00	2.46	0.08	up	0.00	1.52	0.08	up
	xi-2,3-Dihydro-3-methyl furan	0.01	1.60	0.05	down	0.00	1.61	0.05	down
Unknown	13,14-Dihydro-15-keto prostaglandin A2	0.01	2.62	0.05	up	0.00	3.36	0.05	up
	2,3-Dinorthromboxane B1	0.01	1.80	0.15	down	0.00	1.73	0.15	down
	2’-Hydroxy-2,3,5’-trimethoxychalcone	0.00	2.49	0.02	up	0.00	1.73	0.02	up
	Flonicamid	0.00	2.25	0.06	up	0.00	2.42	0.06	up
	Polygodial	0.01	1.25	0.06	down	0.01	2.42	0.06	down

**Table 8:** Comparative regulation (up/down) of the mutually shared metabolites between Q_1/Q_3, Q_1/Q_5, and Q_3/Q_5 groups of the Yunnan semi-fine wool rams fed different levels of dietary calcium.

Superclass	Metabolite	Q_1/Q_3				Q_1/Q_5				Q_3/Q_5			
		*p*-Value	VIP	CV	Regulated	*p*-Value	VIP	CV	Regulated	*p*-Value	VIP	CV	Regulated
Benzenoids	1,1’-[1,12-Dodecanediylbis(oxy)]bisbenzene	0.00	2.72	0.07	up	0.00	1.53	0.07	up	0.00	1.55	0.07	down
	Benzidine	0.00	2.91	0.03	up	0.00	1.71	0.03	up	0.00	1.58	0.03	down
	Citalopram	0.00	2.52	0.10	up	0.00	1.38	0.10	up	0.01	1.47	0.10	down
Lipids and lipid-like molecules	6-Hydroxypentadecanedioic acid	0.01	1.68	0.04	up	0.07	1.19	0.04	down	0.00	3.03	0.04	down
	7alpha-hydroxy-3-oxochol-4-en-24-oic Acid	0.00	2.78	0.08	up	0.01	1.96	0.04	up	0.01	1.85	0.13	down
	Nutriacholic acid	0.00	2.73	0.03	up	0.00	1.66	0.05	up	0.00	1.17	0.05	down
Organic acids and derivatives	N-Phenylacetylglutamic acid	0.00	5.21	0.03	down	0.00	2.75	0.03	down	0.00	3.12	0.03	up
	Phenylpropionate)glycine	0.00	3.31	0.15	down	0.00	2.17	0.05	down	0.00	1.75	0.05	up
Organoheterocyclic compounds	5-Hydroxyindole-3-acetic acid	0.00	2.10	0.19	down	0.00	2.47	0.19	down	0.00	3.04	0.05	down
	Fentanyl	0.00	2.03	0.02	up	0.00	1.88	0.10	up	0.00	1.95	0.02	down
	Indole-3-pyruvic acid	0.00	3.77	0.05	down	0.00	2.01	0.05	down	0.00	2.24	0.05	up
	Quinaldic acid	0.00	1.61	0.04	up	0.00	1.48	0.04	down	0.00	3.24	0.04	down
Unknown	Carbazole	0.00	2.76	0.08	up	0.00	1.44	0.08	up	0.00	1.67	0.08	down

## Data Availability

The data supporting the findings of this study are available on request from the corresponding authors.
